# Pregnancy outcome in a pregnant patient with idiopathic Pulmonary Arterial Hypertension: a case report and review of the literature

**DOI:** 10.1186/s13256-017-1547-1

**Published:** 2018-02-13

**Authors:** Farid Rashidi, Hossein Sate

**Affiliations:** 1 Tuberculosis and Lung Disease Research Center, Tabriz University of Medical Sciences, Imam Reza General Hospital, 29 Bahaman St, Tabriz, Iran; 2 Department of Cardiology, Cardiovascular Research Center, Tabriz University of Medical Sciences, Tabriz, Iran

**Keywords:** Pulmonary hypertension, Pregnancy

## Abstract

**Background:**

Idiopathic pulmonary arterial hypertension is a rare and progressive condition which is aggravated by the physiologic changes during pregnancy. Because of high mortality rate, most physicians recommend early termination of pregnancy in patients with idiopathic pulmonary arterial hypertension.

**Case presentation:**

Here we describe a case of a 30-year-old primigravida Caucasian housewife with functional class 1 idiopathic pulmonary arterial hypertension and a positive vasoreactive response to adenosine who had a full-term non-complicated delivery.

Right-sided heart catheterization before the pregnancy showed severe pulmonary hypertension with mean pulmonary arterial pressure of 60 mmHg, and pulmonary vascular resistance of 12.2 WU. Vasoreactivity was positive after infusion of 200 μg/kg per minute adenosine. During pregnancy, she did not receive medication other than prophylactic enoxaparin. She had an elective cesarean section under general anesthesia at 39 weeks of gestation without complication and delivered a healthy baby. After delivery, her hemodynamic status was stable. One month postpartum, she was in a stable clinical condition in functional class 1.

**Conclusions:**

In pregnant patients with pulmonary arterial hypertension, decreased mortality has been observed over recent years particularly in patients with well-controlled pulmonary pressure and a positive vasoreactivity test.

## Background

Pulmonary arterial hypertension (PAH) is a progressive disease that may affect women of childbearing age. This disease often leads to right ventricular failure and death during pregnancy or after delivery [1, 2]. Some physiological changes during pregnancy can increase pulmonary vascular resistance (PVR) and precipitate right ventricular failure. For these reasons, the mortality rate of PAH during pregnancy is high [3, 4]. However, in recent years, the availability of better care and a multidisciplinary approach have decreased mortality [1, 5, 6]. In this report we describe a 30-year-old pregnant woman with PAH who successfully delivered a healthy baby at 39 weeks of gestation without receiving treatment for pulmonary hypertension during her pregnancy.

## Case presentation

Our patient is a 30-year-old primigravida Caucasian housewife who was referred to a cardiologist because of palpitations. Her medical history was unremarkable. She was not taking any medication and had negative history for tobacco smoking and drinking. Her family history was negative for pulmonary hypertension, systemic hypertension, and diabetes. After getting an echocardiogram, she was diagnosed as having severe pulmonary hypertension. A physical examination revealed that she had a loud pulmonic valve closure (P2) and grade 2/6 systolic murmur on the left sternal border. There was no pitting edema in lower extremities and no clubbing. She had normal pulses in all four extremities. Her oxygen saturation (O_2_ sat) was 91% in room air, and heart rate (HR) was 85 beats per minute at rest. Her blood tests for antinuclear antibodies (ANA), anti-centromere antibodies (Ab), anti-topoisomerase Ab, and human immunodeficiency virus Ab (HIVAb) were all negative. Thyroid function tests—thyroxin (T_4_), triiodothyronine (T_3_), and thyroid-stimulating hormone (TSH)—were normal. She had a normal kidney function test: creatinine (Cr) of 0.7 Mg/dL. Her liver enzymes were normal: aspartate aminotransferase (AST) of 26 U/L and alanine aminotransferase (ALT) of 28 U/L. She had normal urinalysis. In a 6-minute walk test (6MWT) she was able to walk 450 meters without a drop in her O_2_ sat.

She underwent right ventricle catheterization (Table 1). Her vasoreactivity testing with adenosine was positive at 200 ug/minute. She was put on warfarin and amlodipine. In spite of strong advice against pregnancy, she became pregnant 6 months later and stopped taking her medications. In the first prenatal care visit, during the second trimester, she was in good general condition and did not have any complaints including dyspnea. On physical examination, P2 was loud, HR was 89/minute, O_2_ sat was 95%, and there was no edema of lower extremities. In 23rd week of pregnancy she was started on enoxaparin at 40 mg/day subcutaneously. In subsequent visits, her general condition stayed acceptable. She stayed symptom free except for some palpitations during activity. In the third trimester (month 8), she had another echocardiography (Table 2).

**Table 1:** Right-side heart catheterization 6 months before pregnancy

Systemic BP 100/60 (73) mmHg	100/60 (73) mmHg
Heart rate	78/minute
CVP	6 mmHg
Right atrium	8 mmHg
Right ventricular pressure	72/10 (36) mmHg
Pulmonary artery pressure	72/45 (60) mmHg
PAWP	11 mmHg
Cardiac output (thermodilution)	4 liter/minute
Cardiac index	2.31 liter/minute per m^2^
PVR (dyne × second/cm^5^)	980 (12.2 WU)
SVR (dyne × second/cm^5^)	1346 (16.8 WU)
Vasoreactive test (200 μg/kg per minute adenosine)	Mean. PAP = 38 mmHg, cardiac output = 4.2 liter/minute

*BP* blood pressure, *CVP* central venous pressure, *PAP* pulmonary arterial pressure, *PAWP* pulmonary artery wedge pressure, *PVR* pulmonary vascular resistance, *SVR* systemic vascular resistance

**Table 2:** Echocardiography during pregnancy

	26 weeks	37 weeks
IVC	21 mm	22 mm
TAPSE	18 mm	18 mm
TRG	103 mmHg	80 mmHg
RV free wall thickness	10 mm	11 mm
RVSP	113 mmHg	100 mmHg
LVEDD	41 mm	39 mm
LVESD	20 mm	23 mm
Main PA	32 mm	39 mm

*IVC* inferior vena cava, *LVEDD* left ventricular end diastolic diameter, *LVESD* left ventricular end systolic diameter, *PA* pulmonary artery, *RV* right ventricular, *RVSP* right ventricular systolic pressure, *TAPSE* tricuspid annular plane systolic excursion, *TRG* tricuspid regurgitation gradient

In 37th gestational week, enoxaparin was discontinued and our patient was put on subcutaneously administered heparin. She was admitted for elective delivery. On physical examination, her cranial nerves were intact, P2 was loud, blood pressure (BP) was 135/85, HR was 98, and temperature was 37.3 °C. Heparin was stopped 6 hours prior to delivery. She had her third echocardiogram during hospitalization and in the 37th week of gestation (Table 2), which did not show any new findings.

During pregnancy, fetus growth and placenta were normal based on ultrasonography.

She had an elective cesarean section under general anesthesia in her 39th week of gestation. A live, healthy baby boy was delivered. His Apgar score was 9. The baby was 48 cm tall and weighed 2700 gram with head circumference of 32 cm. The baby continued to be healthy 1 year after delivery.

After successful delivery her hemodynamics stayed stable. Heparin infusion was continued and eventually she was bridged to warfarin. After a week, she was transferred to our general ward. She was discharged home 2 weeks after delivery in good condition. One month later, she was seen again. Her general condition was satisfactory, and dyspnea was observed as functional class (FC) 1. At 1-year follow up she was in FC 2. On physical examination, P2 was loud, HR was 78/minute, O_2_ sat was 93%, and there was no edema of lower extremities.

## Discussion

In this case report we presented a 30-year-old primigravida woman with idiopathic PAH (iPAH) who had a successful full-term pregnancy. She did not take medication for pulmonary hypertension during pregnancy and delivered a healthy baby.

Pulmonary hypertension secondary to congenital heart disease or iPAH are important problems among women of childbearing age. Elevated PVR often leads to right ventricular failure and death if left untreated [1, 2]. Pregnancy makes many changes in the maternal cardiovascular system, beginning in the first trimester [7, 8]. These changes increase mortality and morbidity in patients with pulmonary hypertension. Since maternal cardiac output and blood volume can increase by 30 to 50% during the third trimester [7, 8], pregnancy is strongly discouraged in patients with PAH and, if pregnancy occurs, early termination is recommended [9].

Hormonal changes during pregnancy can lead to accumulation of a significant amount of fluid in the interstitial space. Following delivery, this fluid is shifted to the maternal circulation system, causing an increase in preload and hypertension [10]. Furthermore, during labor, uterine contractions could effectively add 500 cc of blood to pulmonary circulation [11]. Also, during vaginal delivery, the Valsalva maneuver and pain can increase HR and vascular resistance. These physiological changes in the presence of pulmonary vascular disease may not be tolerated, and can lead to cardiorespiratory failure [12, 13].

In pregnant women, because of increased prostacyclin production during pregnancy, PVR is approximately 30% less compared to non-pregnant women [1]. This helps better tolerance of large volume load in pulmonary circulation without significant elevation of pulmonary pressure in pregnant women [14]. However, in women with pulmonary hypertension, this normal physiology is impaired. This leads to a further rise in pulmonary artery pressure because of increased cardiac output [15]. Blood volume peaks between the 20th and the 32nd week of gestation [14]. So, the deterioration of pulmonary hypertension during pregnancy occurs after the 20th week of gestation [16, 17]. Usually, delivery is planned between the 32nd and 34th week of the pregnancy [4].

Data suggest that early termination of pregnancy is associated with lower maternal complications and hospital mortality [18]. The mode of delivery and anesthetic management are a matter of debate. In general, increased PVR, marked decrease in venous retention, and myocardial depression should be avoided.

Some of the medication used for anesthesia may influence hemodynamics in patients with pulmonary hypertension [19, 20]. Many physicians do not suggest cesarean section as a delivery method in these patients [21]. Vaginal delivery is usually associated with reduced risk of bleeding and infection, but cesarean section precludes prolonged labor and allows for the careful consideration of optimal anesthesia and its administration. Regional anesthesia is generally preferred over general anesthesia because regional anesthesia has less effect on systemic vascular tone and cardiac function [22]. Maternal mortality may increase with general anesthesia [21]. Approximately 70 to 75% of the patients are delivered under epidural anesthesia [23]. Since our patient had stable hemodynamics at her 37th week of pregnancy, we decided to delay the delivery until week 39. The decision regarding time and delivery method depends on maternal hemodynamics and fetal health. An elective cesarean section under general anesthetic was planned and delivery took place without any complications. This is a rare case of iPAH with successful delivery after 38 weeks of pregnancy. Until the past decade, pregnant patients with PAH had a very high mortality rate and patients with PAH were strongly advised against pregnancy. In recent years, these patients experienced less mortality. A retrospective review study showed a 30% mortality rate in pregnant patients with iPAH, 36% in patients with Eisenmenger syndrome, and 56% in patients with PAH associated with other conditions [23]. Another study which was published later, showed less mortality [21]. In the later study many patients were treated with pulmonary vasodilators. A more recent study involving 26 women with PAH who were treated at 13 pulmonary hypertension centers from 2007 to 2010 showed high mortality but the mortality was lower than that found in former studies [24]. It seems one of the major reasons for less mortality in the most recent study was PAH targeted therapy. In this study, the patients who died had very high PVR: from 1560 to 1928 dyn.second.cm^−5^. Patients with vasoreactive pulmonary hypertension had a better prognosis during pregnancy [25].

Our patient had pulmonary hypertension with positive vasoreactivity. Also, she was in FC I with PVR around 980 dyn.second.cm^−5^. During pregnancy, she was well in FC I without any hemodynamic instability. She underwent cesarean section under general anesthesia in her 39th week of pregnancy and delivered a healthy baby. In previous studies, most patients delivered after 34 weeks [26].

## Conclusions

Based on studies during the past three decades, the risk of mortality in pregnant patients with PAH remains high. There is recent evidence showing reduced mortality, particularly in patients with well-controlled pulmonary pressure, low PVR, and positive vasoreactivity test results.

Nonetheless, because of limited data, there is no general agreement about the safety of pregnancy even in patients with well-controlled pulmonary hypertension. Overall, pregnancy should be discouraged in this population. Early termination of pregnancy should be considered in patients with severe PAH.
